# Two new species of *Leptopsyllus* from Korea (Copepoda, Harpacticoida, Paramesochridae)

**DOI:** 10.3897/zookeys.665.6150

**Published:** 2017-04-03

**Authors:** Jinwook Back, Wonchoel Lee

**Affiliations:** 1 Department of Taxonomy and Systematics, National Marine Biodiversity Institute of Korea, Seocheon, 325-902, Korea; 2 Department of Life Science, College of Natural Sciences, Hanyang University, Seoul 133-791, Korea

**Keywords:** Copepoda, Harpacticoida, Korea, *Leptopsyllus*, Paramesochridae, taxonomy

## Abstract

Two new species of *Leptopsyllus* are described from the subtidal zone of Korea. Both species were assigned to the subgenus Leptopsyllus (Leptopsyllus) T. Scott, 1894, based on following three characters: two-segmented rami of P1, absence of endopod on P2 and P3, and presence of one-segmented endopod of P4. L. (L.) pundius **sp. n.** is most closely related to L. (L.) punctatus Mielke, 1894, however clearly distinguishable from it based on mandibular exopod with two setae, shape of P6, and caudal seta III ornamented with spinules in the new species. L. (L.) koreanus **sp. n.** is clearly distinguishable from its congeners by the second segment of P1 endopod armed with one element, male baseoendopod of P5 with one seta, and one segmented endopod of mandibular palp. The world distribution and updated key to the species of the genus *Leptopsyllus* are provided.

## Introduction

Although the marine biodiversity of Korea is very high according to a recent estimation (Costello et al. 2010), many small interstitial organisms, including harpacticoid copepods, still remain unknown. Harpacticoid copepods play an important role in the benthic food web, and are an important source of biodiversity in Korea. Thus far, 88 harpacticoid species (58 genera and 23 families) including planktonic, free-living benthic, and invertebrate-associated species have been reported from Korean waters (Song et al. 2012).

Paramesochrid harpacticoids, with their reduced appendages and vermiform body shapes, successfully inhabit subtidal and intertidal sandy bottoms (Boxshall and Halsey 2004). Many free-living genera of the family Paramesochridae have adapted to living on various sandy sediments irrespective of depth and salinity (Plum and George 2009). For example, *Emertonia clausi* Pointer & Veit-Köhler, 2013 was collected from the deep sea (Pointner et al. 2013) while *Remanea naksanensis* Back, Lee & Huys, 2011 was collected from brackish water (Back et al. 2011). So far, nine species belonging to four genera in the family Paramesochridae have been discovered in Korea (Back and Lee 2014).

Thomas Scott (1894) proposed the genus *Leptopsyllus* and fixed *Leptopsyllus typicus* T. Scott, 1894 as the type and presented a generic diagnosis and description of this species. Lang (1944) created the new genus *Paraleptopsyllus* based on the one-segmented P3 endopod. In Kunz’s (1962) revision of the family Paramesochridae, the author proposed nine genera including four new genera, *Apodopsyllus*, *Intermedopsyllus* (accepted as Wellsopsyllus (Intermedopsyllus) Huys, 2009), *Kliopsyllus* Kunz, 1962 (accepted as *Emertonia* Wilson, 1932), and *Scottopsyllus* Kunz, 1962 (accepted as Wellsopsyllus (Scottopsyllus) Apostolov & Marinov, 1988), based on the segmentation of the legs. Subsequently, each species originally placed in *Leptopsyllus* was allocated to a suitable genus. However, Kunz (1981) revised the family Paramesochridae and treated the genus *Paraleptopsyllus* as subgenus. Especially, Huys (2009) proposed the correcting name and authorship of subgenus Intermediopsyllus Huys, 2009 in accordance with ICZN Art.16, because Kunz (1962) contravened ICZN Art.13.3. As a result, the genus *Leptopsyllus* comprises two subgenera, *Leptopsyllus* T. Scott, 1894 and *Paraleptopsyllus* Lang, 1944 and the genus *Leptopsyllus* currently consists of 11 valid species (Wells 2007). Until now, only one species, Leptopsyllus (Paraleptopsyllus) arcticus (Lang, 1936), has been assigned to the subgenus Paraleptopsyllus.

A survey of harpacticoid copepods from subtidal zones in Korea resulted in the discovery of two new species belonging to Leptopsyllus (Leptopsyllus). Here these two species are described and an updated key to species of the genus is provided.

## Materials and methods

Specimens were collected from sediments in the subtidal zone near Pung Island off the west coast of Korea (Leptopsyllus (Leptopsyllus) pundius sp. n.), and Maemul Island (Leptopsyllus (Leptopsyllus) koreanus sp. n.) off the south coast of Korea. Sediments were collected using a grab (surface area 0.1 m^2^) and fixed with 5% buffered formalin. Copepods were extracted from the sediment samples using the Ludox method (Burgess 2001) and preserved in 70% ethanol. Dissected specimens were mounted on several slides separately using lactophenol as a mounting medium. Slides were sealed with transparent nail varnish. Observations were made using a microscope (Olympus BX51) equipped with differential interference contrast and a drawing tube.

The descriptive terminology of Huys et al. (1996) was adopted. Abbreviations used in the descriptions are:

**A1** antennule;

**A2** antenna;

**ae** aesthetasc;

**exp** exopod;

**enp** endopod;

**P1–P6** first to sixth thoracopod;

**exp (enp)-1 (2, 3)** to denote the proximal (middle, distal) segment of a three-segmented ramus;

**CR** caudal ramus.

Specimens were deposited in the National Marine Biodiversity Institute of Korea (**MABIK**). Scale bars in figures are in μm.

## Systematics

### Family Paramesochridae Lang, 1944

#### Genus Leptopsyllus (Leptopsyllus)

Taxon classificationAnimaliaHarpacticoidaParamesochridae

T. Scott, 1894

##### Updated genus diagnosis.

Paramesochridae. Body cylindrical, depressed dorsoventrally; with distinct separation between prosome and urosome; rostrum fused with cephalothorax. Caudal ramus with 5–7 setae. Antennule 7- or 8-segmented in female, subchirocer in male. Antennary exopod 1-segmented (except for L. (L.) dubatyi, 2-segmented) with 3–5 setae. Maxilla with 3 endites on syncoxa; endopod 1-segmented. Maxilliped with elongate basis; endopod 1- or 2-segmented. P1 biramous, 2-segmented endopod and exopod. P2 uniramous; without endopod; with 3-segmented exopod, except for L. (L.) abyssalis with 2-segmented exopod. P3 uniramous; without endopod, except for L. (P.) arcticus with 1-segmented endopod; with 3-segmented exopod, except for L. (L.) abyssalis with 2-segmented exopod. P4 biramous; with 1-segmented endopod; with 3-segmented exopod, except for L. (L.) abyssalis with 2-segmented exopod. P1–P4 armature formulae:

**Table:** 

	Exopod	Endopod
P1	0.022	0.020
P2	0[1]^1^.0[1]^2^.011	
P3	0.0[1]^2^.011	
P4	0.0.011	010

^1^ L. (L.) paratypicus ^2^ L. (L.) paratypicus, L. (L.) celticus

Exopod of P5 armed with 3 setae in both sexes.

Sexual dimorphisms in A1, P5, P6 and genital segment.

##### Type species.

Leptopsyllus (Leptopsyllus) typicus T. Scott, 1984

##### Additional species.

L. (L.) paratypicus Nicholls, 1939; L. (L.) reductus Lang, 1948; L. (L.) harveyi Wells, 1963; L. (L.) elongatus Drzycimski, 1967; L. (L.) dubatyi Soyer, 1974; L. (L.) abyssalis Becker, Noodt & Schriever, 1979 ; L. (L.) platyspinosus Mielke, 1984; L. (L.) punctatus Mielke, 1984; L. (L.) celticus Bodin & Jackson, 1987; L. (L.) pundius sp. n.; and L. (L.) koreanus sp. n.

#### Leptopsyllus (Leptopsyllus) pundius sp. n.

Taxon classificationAnimaliaHarpacticoidaParamesochridae

http://zoobank.org/D56A6D69-3919-4719-899A-889C1023E935

Figs 1 , 2 , 3 , 4

##### Type locality.

Republic of Korea, Pung Island (Korean name Pungdo): subtidal zone, 37°5'21.46"N, 126°24'27.10"E (depth: 30 m, sand).

##### Materials examined.

Holotype 1♀ (MABIK CR00235287) dissected on four slides. Sampled by a grab on a fishing boat on 16 Feb 2008.

##### Diagnosis.

**Figure 1.**
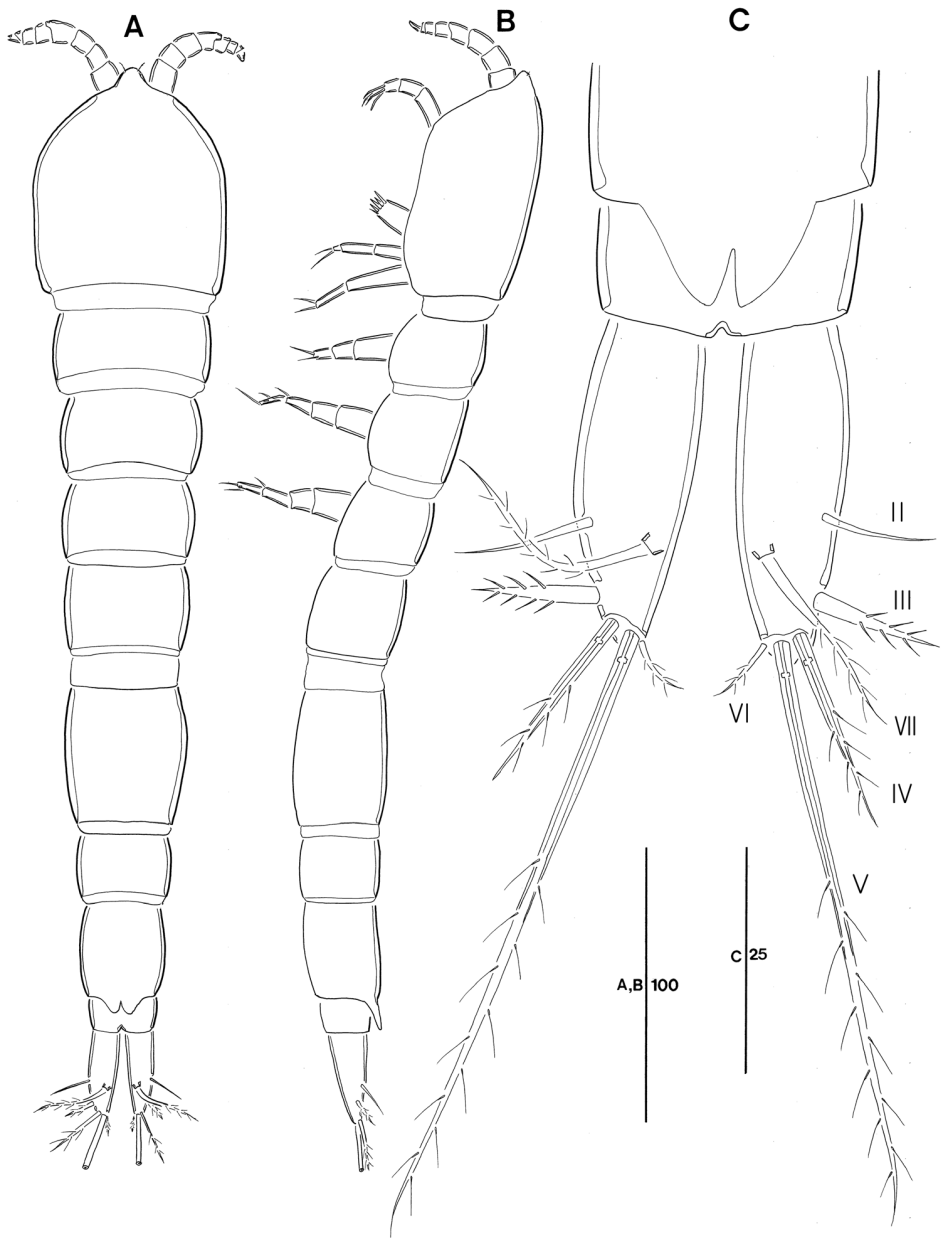
Leptopsyllus (Leptopsyllus) pundius sp. n. (♀). **A** habitus, dorsal **B** habitus, lateral **C** pseudoperculum, anal somite, and caudal rami, dorsal. Scale bars are in microns.

**Description of female.** Total body length 390 µm (Fig. 1A); largest width measured at posterior margin of cephalic shield: 67 µm; body cylindrical, slightly depressed dorsoventrally; urosome gradually tapering posteriorly; whole body very hyaline. Rostrum triangular, fused with cephalic shield; with 2 small sensilla. Cephalothorax bell-shaped; pleural areas weakly developed and posterolateral angles rounded; posterior margin smooth, without distinct hyaline frill.

**Figure 2.**
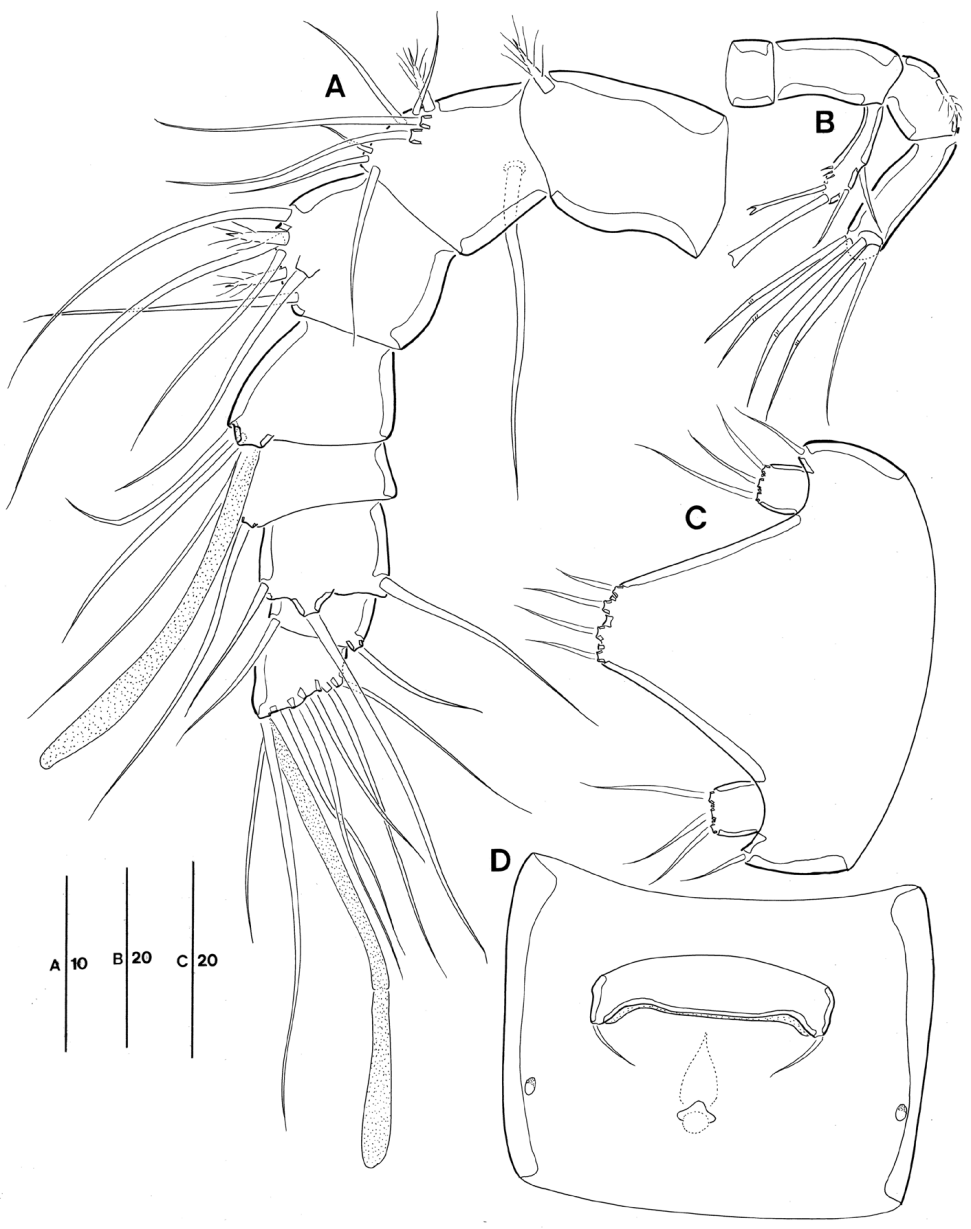
Leptopsyllus (Leptopsyllus) pundius sp. n. (♀). **A** antennule **B** antenna **C** P5 **D** P6 and genital field. Scale bars are in microns.

*Genital field* located mid-ventrally halfway the length of the genital double-somite; copulatory pore located near posterior border of genital field and covered by small process (Fig. 2D); P6 represented by transverse plate with 1 bare seta (Fig. 2D); penultimate somite with well-developed pseudoperculum; anal operculum not developed.

*CR* (Fig. 1C). Parallel, about 2.7–3.0-times as long as greatest width, conical, distal margin blunt; each ramus armed with 6 setae (seta I not observed and probably vestigial); seta II bare; setae III stout, bearing spinule-like elements; seta IV pinnate; seta V pinnate, longest; seta VI shortest and pinnate; seta VII bi-articulate at base and arising from inner dorsal surface.

*A1* (Fig. 2A). 8-segmented, short, robust; seg-1 longest; seg-4 sub-cylindrical process armed with long slender seta fused basally to aesthetasc; seg-6 armed with 1 slender bare seta arising from ventral sub-cylindrical process; armature formula: 1–[1 pinnate], 2–[8 bare + 1 pinnate], 3–[5 bare + 2 pinnate], 4–[2 bare + (1 + ae)], 5–[1 bare], 6–[3 bare], 7–[2 bare], 8–[5 bare + acrothek]; apical acrothek consisting of well-developed aesthetasc fused basally to 2 slender, naked setae.

*A2* (Fig. 2B). 4-segmented, comprising coxa, basis, and free 2-segmented enp; coxa small and bare; basis approximately 2.2 times as long as maximum width, without any surface ornamentation; exp 1-segmented, with 2 lateral bare and 2 distal modified setae; proximal endopodal segment with 1 pinnate abexopodal seta; distal endopodal segment armed with 2 bare short spines laterally, 1 naked and 2 spine-like setae in middle of segment, 3 geniculate setae around distal margin, and 1 longest geniculate seta fused at base with 1 bare seta.

**Figure 3.**
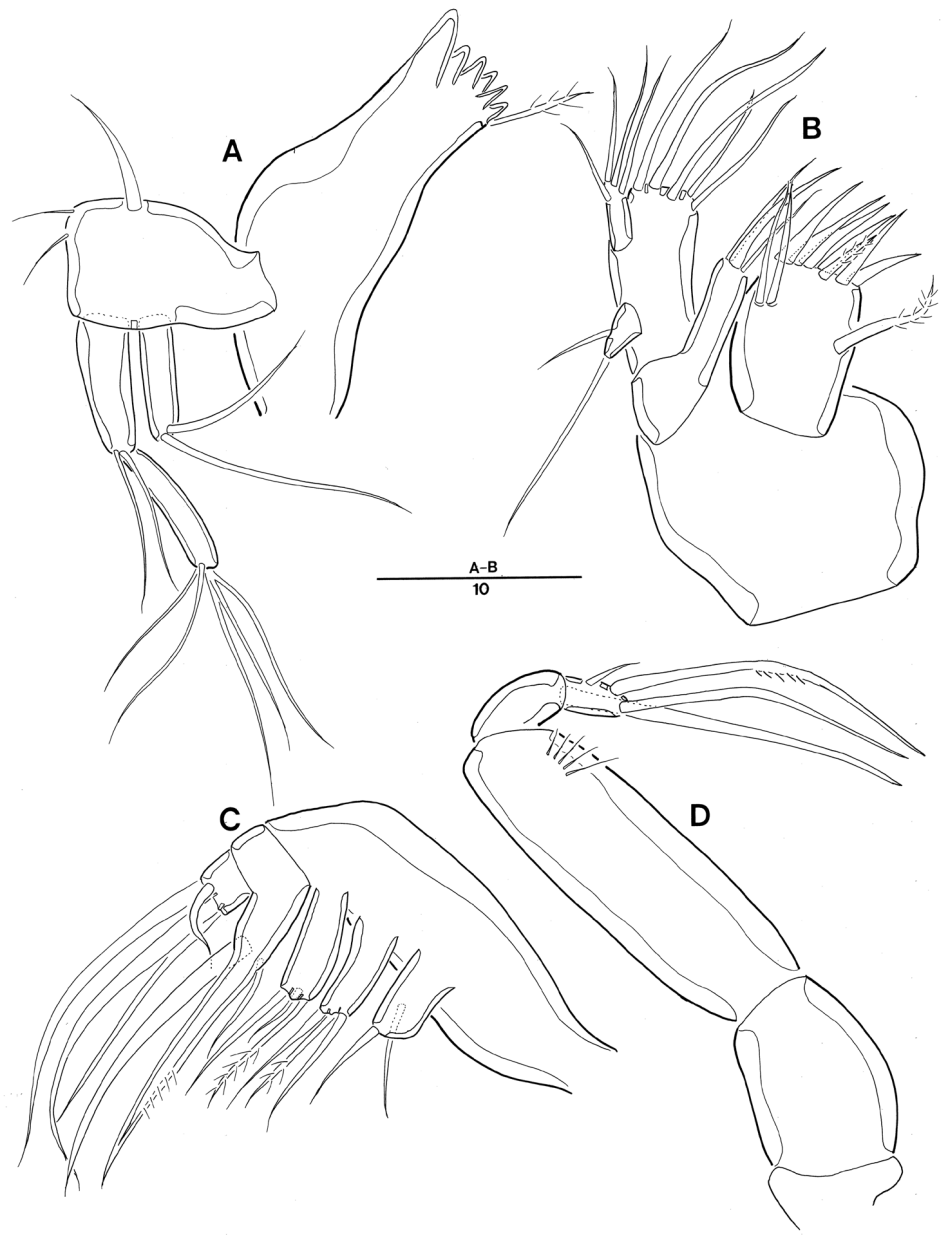
Leptopsyllus (Leptopsyllus) pundius sp. n. (♀). **A** mandible **B** maxillule **C** maxilla **D** maxilliped. Scale bars are in microns.

*Mandible* (Fig. 3A). Coxa with well-developed gnathobase bearing 1 pinnate seta at dorsal corner and 6 major spinous overlapping teeth; palp biramous, comprising basis, 1-segmented exp and 2-segmented enp; basis widening distally, with 3 bare setae; exp with 2 distal setae; enp long; enp-1 same as long as exp, with 2 bare setae; enp-2 with 5 basally fused setae at apex.

*Maxillule* (Fig. 3B). Praecoxal arthrite well-developed, with 6 spines, 2 pinnate setae, and 2 juxtaposed slender setae; coxa with cylindrical endite bearing 3 distal bare setae; basis cylindrical; endites fused, collectively bearing 5 distal bare setae; exp 1-segmented, small, with 2 bare setae; enp 1-segmented, with 4 bare setae distally.

*Maxilla* (Fig. 3C). Syncoxa with 3 cylindrical endites; praecoxal endite with 2 bare setae; proximal and distal coxal endite with 1 pinnate and 2 bare setae each; allobasis with 2 strong claws and 1 bare seta; enp 1-segmented, with 5 bare setae apically.

*Maxilliped* (Fig. 3D) comprising syncoxa, basis and 2-segmented enp; syncoxa without element; basis with 1 row of spinules sub-distally; enp-1 with 1 stout seta on distal margin; enp-2 with 1 bare and 2 geniculate setae.

**Figure 4.**
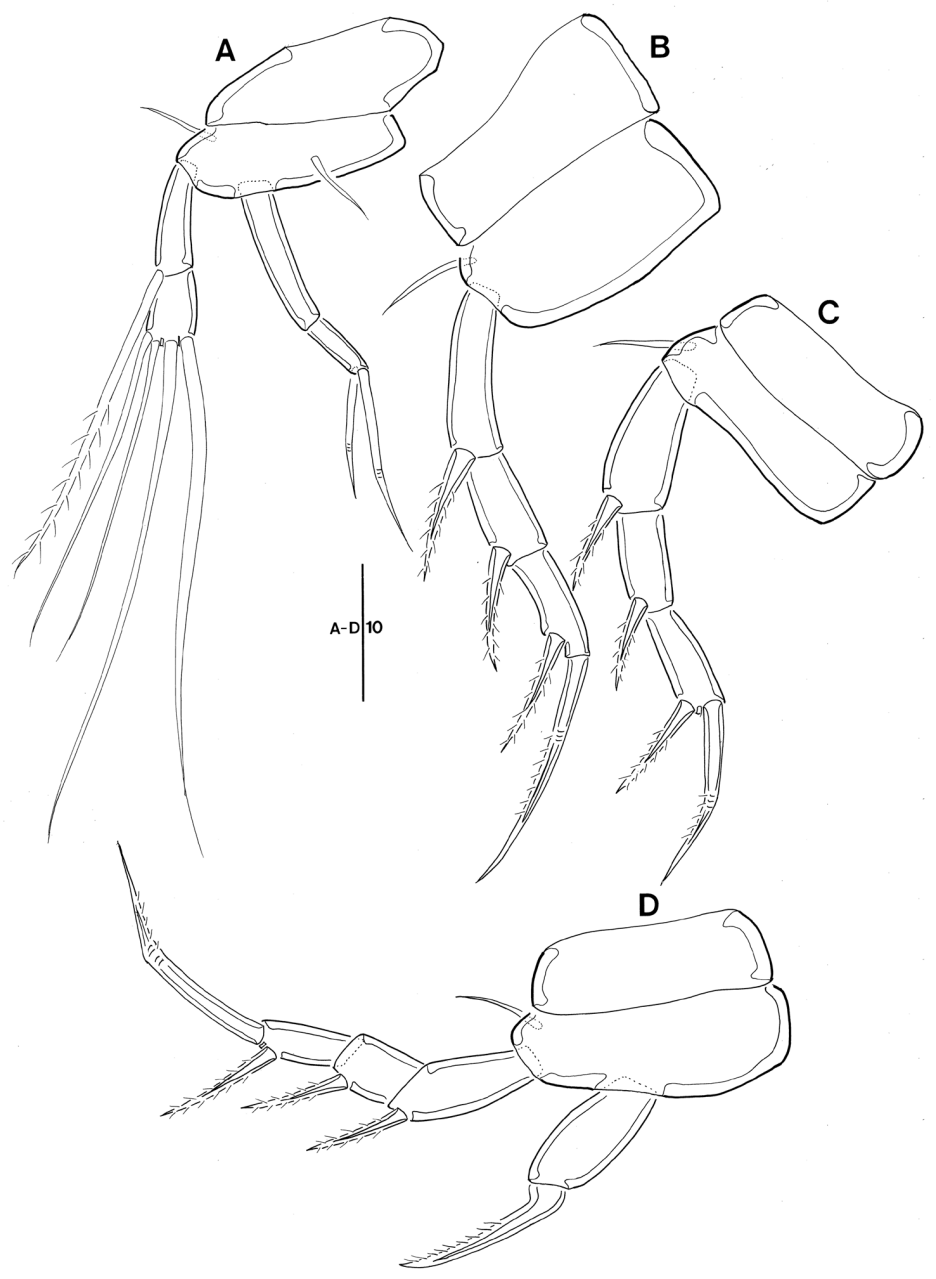
Leptopsyllus (Leptopsyllus) pundius sp. n. (♀). **A** P1 **B** P2 **C** P3 **D** P4. Scale bars are in microns.

*P1* (Fig. 4A). Coxa bare; basis with 1 bare seta on proximal inner margin and 1 small bare outer seta; exp shorter than enp; exp-1 about 1.6 times longer than exp-2, with 1 pinnate seta near outer distal corner; exp-2 with 4 long bare setae distally; enp-1 unornamented, elongate, and approximately 2.3 times as long as enp-2; enp-2 small, with 2 geniculate setae apically.

*P2, P3* (Fig. 4B, C). Coxa bare; basis without any surface ornamentation; outer margin with 1 bare seta; exp 3-segmented; exp-1 and -2 with 1 outer pinnate spine; exp-3 with 1 pinnate outer spine and 1 geniculate seta; enp absent.

*P4* (Fig. 4D). Coxa bare; basis with 1 bare seta; exp 3-segmented; exp-1 and -2 with 1 outer spine; exp-3 with 1 outer spine and 1 geniculate seta; enp as long as exp-1, with 1 strong spine distally.

Armature formula as follows:

**Table:** 

	Exopod	Endopod
P1	0.022	0.020
P2	0.0.011	
P3	0.0.011	
P4	0.0.011	010

P5 (Fig. 2C) with medially fused baseoendopods and discrete exps; baseoendopod with 1 basal seta; endopodal lobes elongate, closely pressed to each other, with 2 bare apical setae each; exp small, with 3 bare setae, innermost one longest.

**Description of male.** Unknown.

##### Etymology.

The specific name refers to the type locality of the new species, Pung Island, Korea.

#### Leptopsyllus (Leptopsyllus) koreanus sp. n.

Taxon classificationAnimaliaHarpacticoidaParamesochridae

http://zoobank.org/98FAF7F8-CC51-4EAF-9D2D-6EF13672A363

Figs 5 , 6 , 7 , 8

##### Type locality.

Republic of Korea, Maemul Island (Korean name : Maemuldo), subtidal zone off 37° 37'43.38"N, 128° 46'24.51"E (depth: 50 m, muddy sand).

##### Material examined.

Holotype 1♂ (MABIK CR00235288) dissected on four slides. Sampled by a grab on a fishing boat on 23 Feb 2011.

##### Diagnosis.

**Description of female.** Unknown.

**Figure 5.**
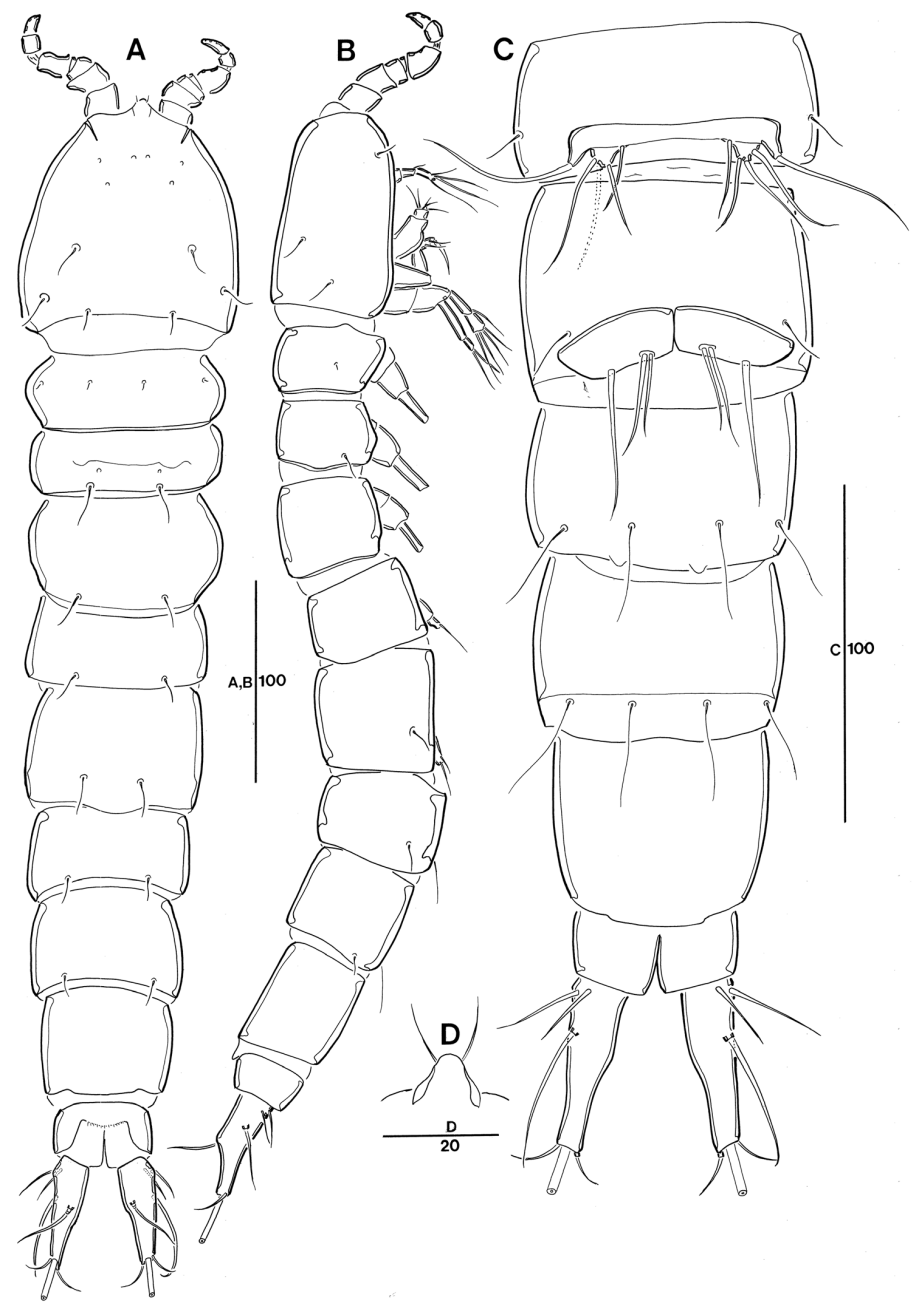
Leptopsyllus (Leptopsyllus) koreanus sp. n. (♂). **A** habitus, dorsal **B** habitus, lateral **C** urosome, ventral **D** rostrum. Scale bars are in microns.

**Figure 6.**
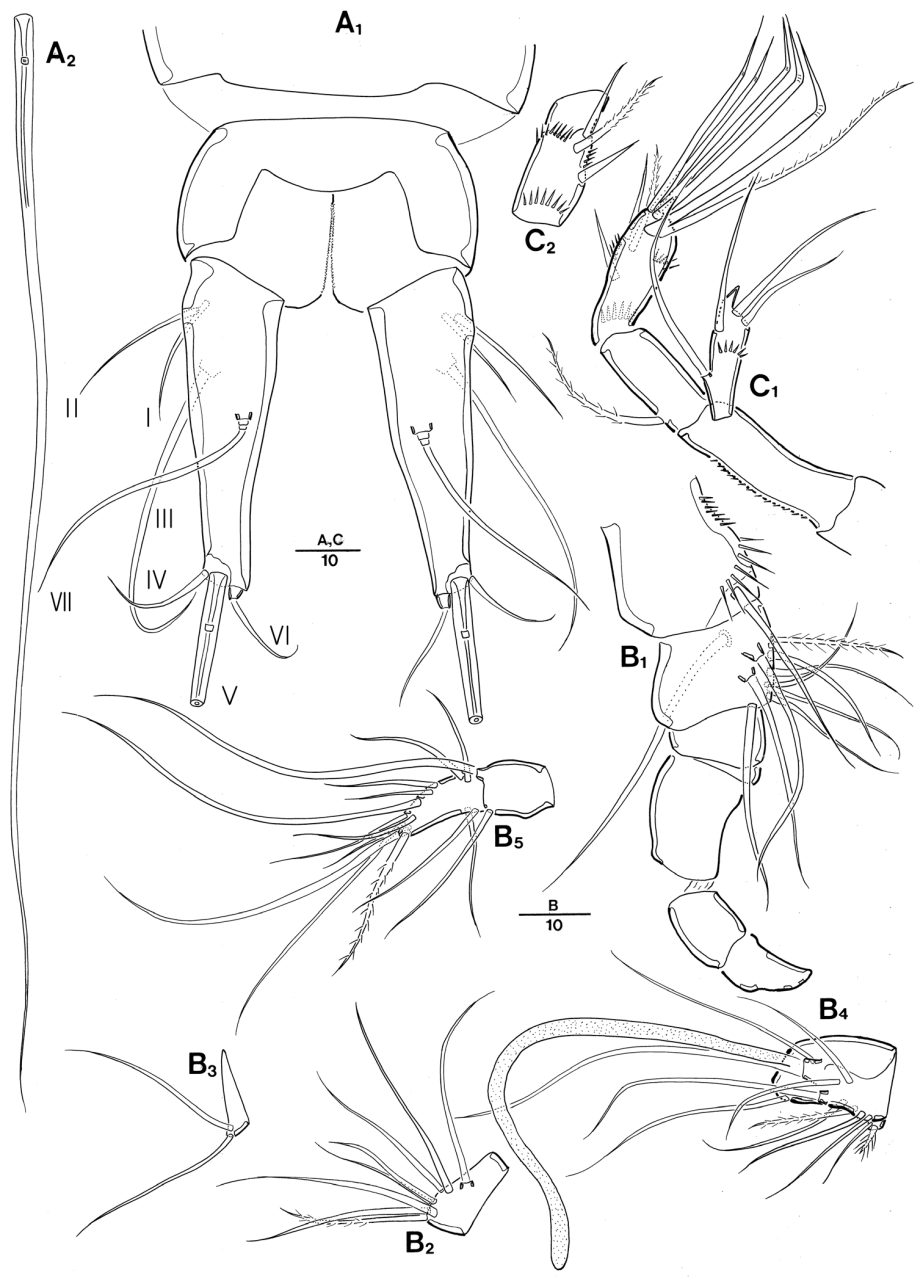
Leptopsyllus (Leptopsyllus) koreanus sp. n. (♂). **A_1_** anal somite and caudal ramus, dorsal **A_2_** seta V **B_1_** segments-1 and -2 of antennule **B_2_** segment-3 **B_3_** segment-4 **B_4_** segment-5 **B_5_** segments-6 and -7 **C_1_** antenna **C_2_** endopod-2 of antenna, lateral. Scale bars are in microns.

**Description of male.** Total body length 575 µm; largest width measured at posterior margin of cephalic shield: 105 µm (Fig. 5A); body cylindrical and slightly depressed dorsoventrally; urosome gradually tapering posteriorly; sensilla present as illustrated in Fig. 5A–C; body somites connected by well-developed arthrodial membranes. Rostrum small, fused with cephalic shield; with 2 sensilla (Fig. 5D). Cephalothorax (Fig. 5A, B) bell-shaped, smooth posterior margin, with few sensilla; pleural areas weakly developed and posterolateral angles rounded; posterior margin smooth, without distinct hyaline frill. Anal somite (Figs 5A, 6A_1_) with developed spinulose operculum.

*CR* (Fig. 6A_1_, A_2_). Parallel, about 3.1-times as long as greatest width, conical, distal margin rounded; each ramus armed with 7 setae; seta I bare, situated rather ventrally; setae II and III bare, situated laterally; seta IV shortest, bare; seta V longest, about 3 times as long as the caudal ramus; seta VI bare, composite, consisting of proximal process and distal seta; setae IV–VI displaced onto dorsal surface of ramus; seta VII tri-articulate at base and arising from inner dorsal surface.

*A1* (Fig. 6B_1_–B_5_) 7-segmented, short, robust, subchirocer; seg-1 with row of spinules along sub-distal margin; seg-5 swollen; armature formula: 1–[1 bare], 2–[9 bare + 1 pinnate], 3–[7 bare + 1 pinnate], 4–[2 bare], 5–[9 bare + 2 pinnate + (1 + ae)], 6–[2 bare], 7–[12 bare + 1 pinnate]; visible apical acrothek not present .

*A2* (Fig. 6C_1_, C_2_) 4-segmented, comprising coxa, basis, 2-segmented enp, and 1-segmented exp; coxa small and bare; basis approximately 3.1-times as long as maximum width, ornamented with row of spinules along inner margin; exp inner distal corner forming spinous projection with 2 lateral and 2 distal naked setae; proximal endopodal segment with 1 pinnate abexopodal seta; distal endopodal segment ornamented with 2 rows of spinules horizontally, with 2 spine-like setae, 1 pinnate seta sub-apically (Fig. 6C_2_), 4 geniculate setae around distal margin, and 1 longest geniculate seta fused at base with 1 longest seta.

**Figure 7.**
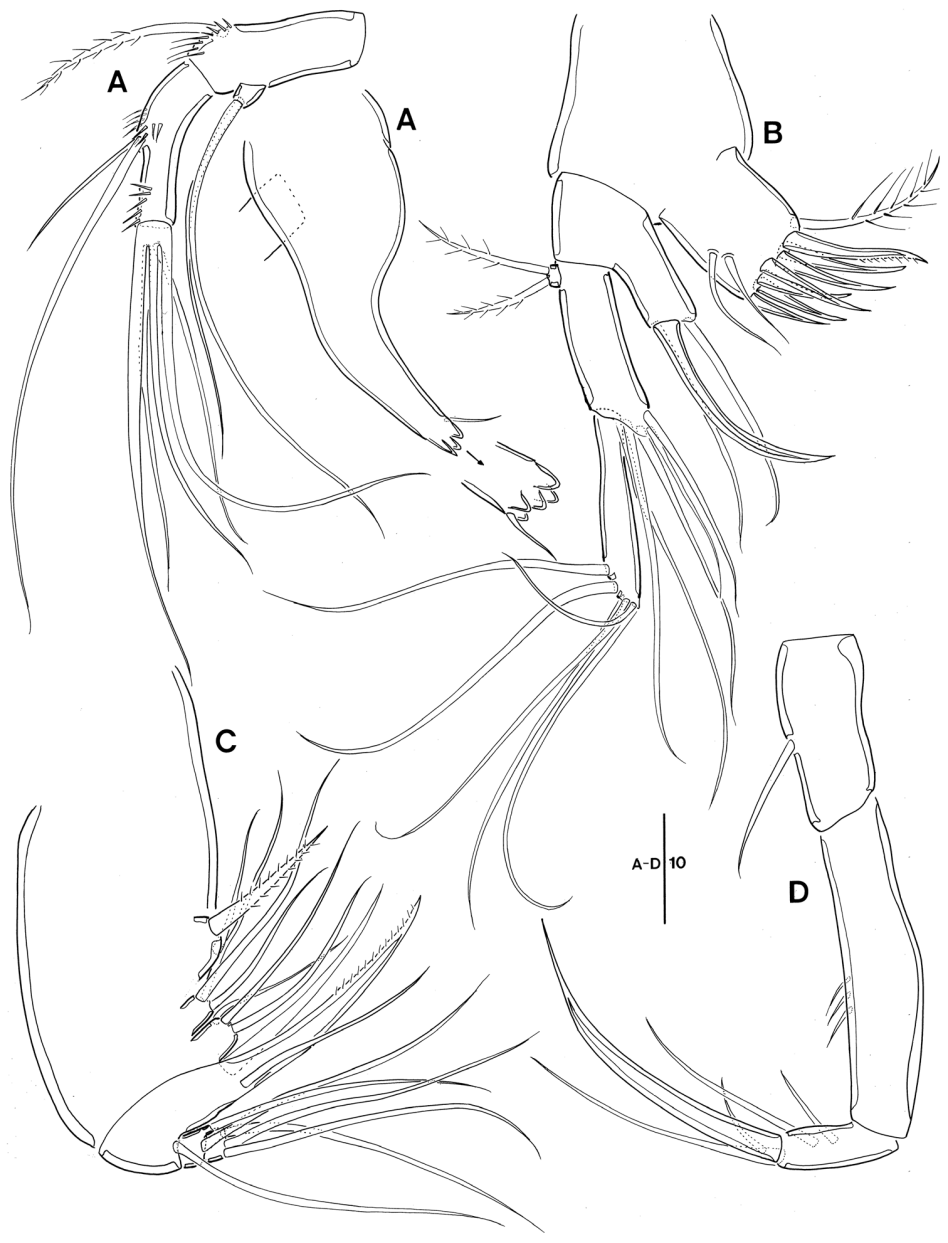
Leptopsyllus (Leptopsyllus) koreanus sp. n. (♂). **A_1_** gnathobase of mandible **A_2_** mandibular palp **B** maxillule **C** maxilla **D** maxilliped. Scale bars are in microns.

*Mandible* (Fig. 7A_1_, A_2_). Coxa with well-developed gnathobase bearing 1 bare seta at the dorsal corner and 6 overlapping teeth; palp biramous, comprising basis, 1-segmented exp and enp; basis with 1 pinnate seta and ornamented with row of spinules near base of seta; exp small, with 2 bare setae; enp long with 2 lateral setae in middle and 5 basally fused setae at apex.

*Maxillule* (Fig. 7B). Praecoxal arthrite well-developed, with 7 spines, 1 pinnate seta, and 2 juxtaposed slender setae on anterior surface; coxa with cylindrical endite bearing 1 claw and 2 naked setae; basis cylindrical; endites fused, with 5 naked setae; exp 1-segmented, small, with 2 pinnate setae; enp 1-segmented, elongate, rectangular, with 6 naked setae around apex.

*Maxilla* (Fig. 7C). Syncoxa with 3 endites; praecoxal endite with 1 pinnate and 1 naked setae; proximal and distal coxal endite with 3 naked setae; allobasis with 1 uni-pinnate strong claw, 1 bare claw, 1 accessory seta, and 1 bare seta near base of enp; enp 2-segmented; enp-1 with 2 bare setae; enp-2 with 3 bare setae apically.

*Maxilliped* (Fig. 7D) 3-segmented, comprising syncoxa, basis and 1-segmented endopod; syncoxa with 1 bare seta; elongate basis ornamented with 3 spinules in middle; enp 2.5 times as long as wide, with 2 naked seta laterally, 1 apical seta, 1 curved stout claw, and 1 accessory on claw.

**Figure 8.**
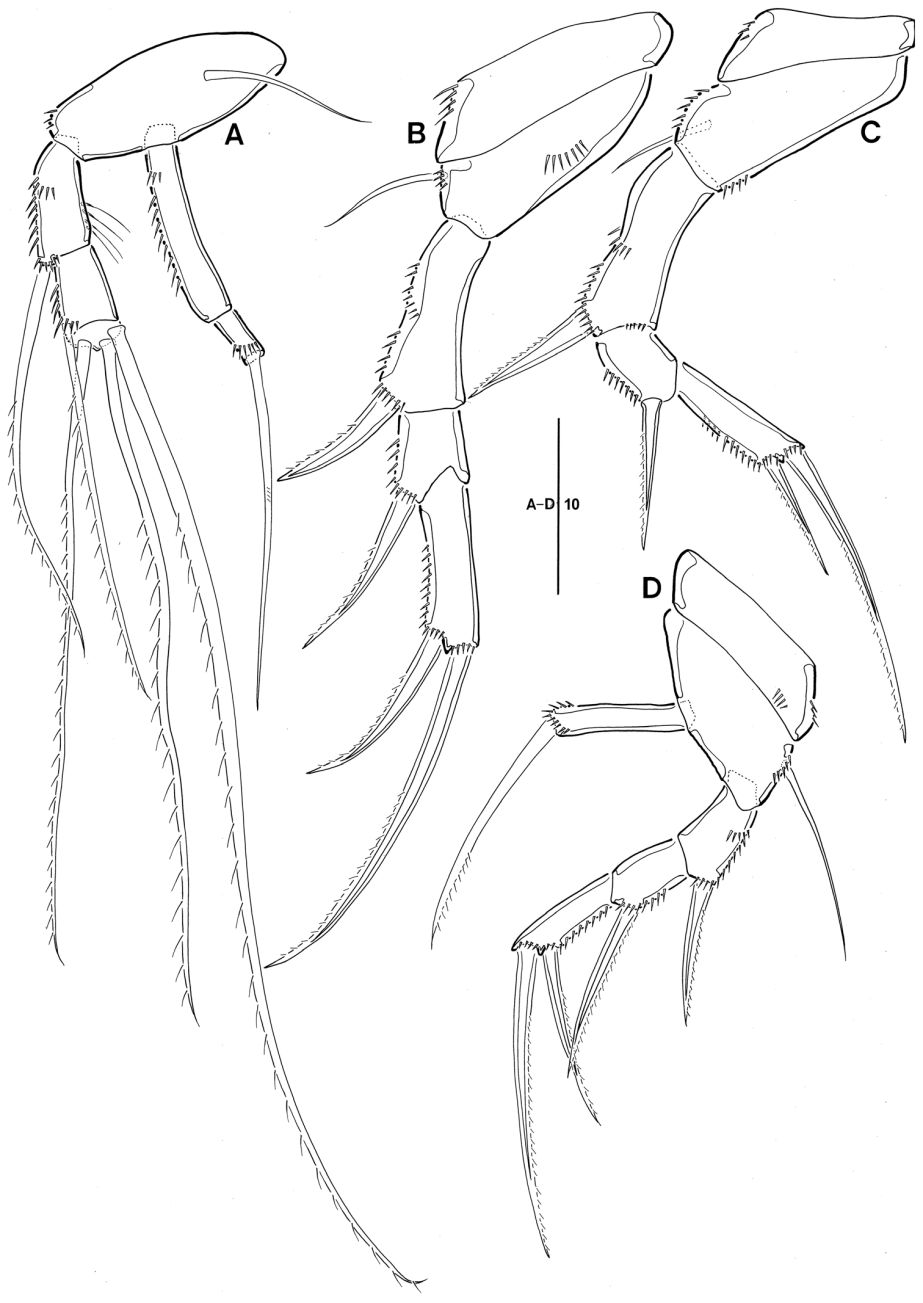
Leptopsyllus (Leptopsyllus) koreanus sp. n. (♂). **A** P1 **B** P2 **C** P3 **D** P4. Scale bars are in microns.

*P1* (Fig. 8A). Basis without outer seta, with 1 bare seta on proximal inner margin; exp shorter than enp; exp-1 with 1 long uni-pinnate seta near outer distal corner, ornamented with row of spinules along outer margin and with long spinules on inner margin; exp-2 with 4 long uni-pinnate setae; enp-1 elongate and approximately 3.5 times as long as enp-2 and ornamented with row of spinules along outer margin; enp-2 small, with 1 geniculate seta.

*P2, P3* (Fig. 8B, C). Coxa ornamented with rows of spinules as figured; basis with 1 outer bare seta and ornamented with row of spinules on inner and outer margin; exp 3-segmented; exp-1 longest, with 1 outer uni-pinnate spine; exp-2 shortest, with 1 outer uni-pinnate spine; exp-3 sub-rectangular, with 2 pinnate spines; enp absent.

*P4* (Fig. 8D). Coxa ornamented with 2 rows of spinules; basis with 1 outer seta; exp 3-segmented; exp-1 and -2, with 1 outer uni-pinnate spine; exp-3 with 2 uni-pinnate spines; enp represented by elongate segment with 1 spine-like seta.

Armature formula as follows:

**Table:** 

	Exopod	Endopod
P1	0.022	010
P2	0.0.011	
P3	0.0.011	
P4	0.0.011	010

*P5* (Fig. 5C). Exopod and baseoendopod not fused; baseoendopod with 1 outer basal seta, endopodal lobes confluent with 1 seta each; exp small, triangular, with 3 naked setae.

*P6* (Fig. 5C) symmetrical, with 2 bare setae arising from small protrusion on inner part of P6, and 1 outer longest bare seta.

##### Etymology.

The specific name refers to the type locality of the new species in Korea.

## Discussion

### General status and zoogeography of the genus *Leptopsyllus* T. Scott, 1894

Since the genus *Leptopsyllus* was proposed by Scott T (1894) based on the reduction of legs, several species have been described in *Leptopsyllus*. Though many species were originally assigned to the genus *Leptopsyllus*, some of them were moved to new genera according to new classifications based on leg characteristics (reduced or absent). Boxshall and Halsey (2004) proposed the number of species in each genus and the key to genera based on Kunz (1981), Huys (1987), and Cottarelli and Forniz (1994). As a result, the genus *Leptopsyllus* currently consists of 13 valid species including the two new species described in this study. However, the complete descriptions of mouthparts are lacking for many species, because of the small body size of these organisms. In addition, the abundant of species in *Leptopsyllus* is usually low. Unfortunately, we founded only one female of L. (L.) pundius sp. n. and one male of L. (L.) koreanus sp. n. during the study.

**Figure 9.**
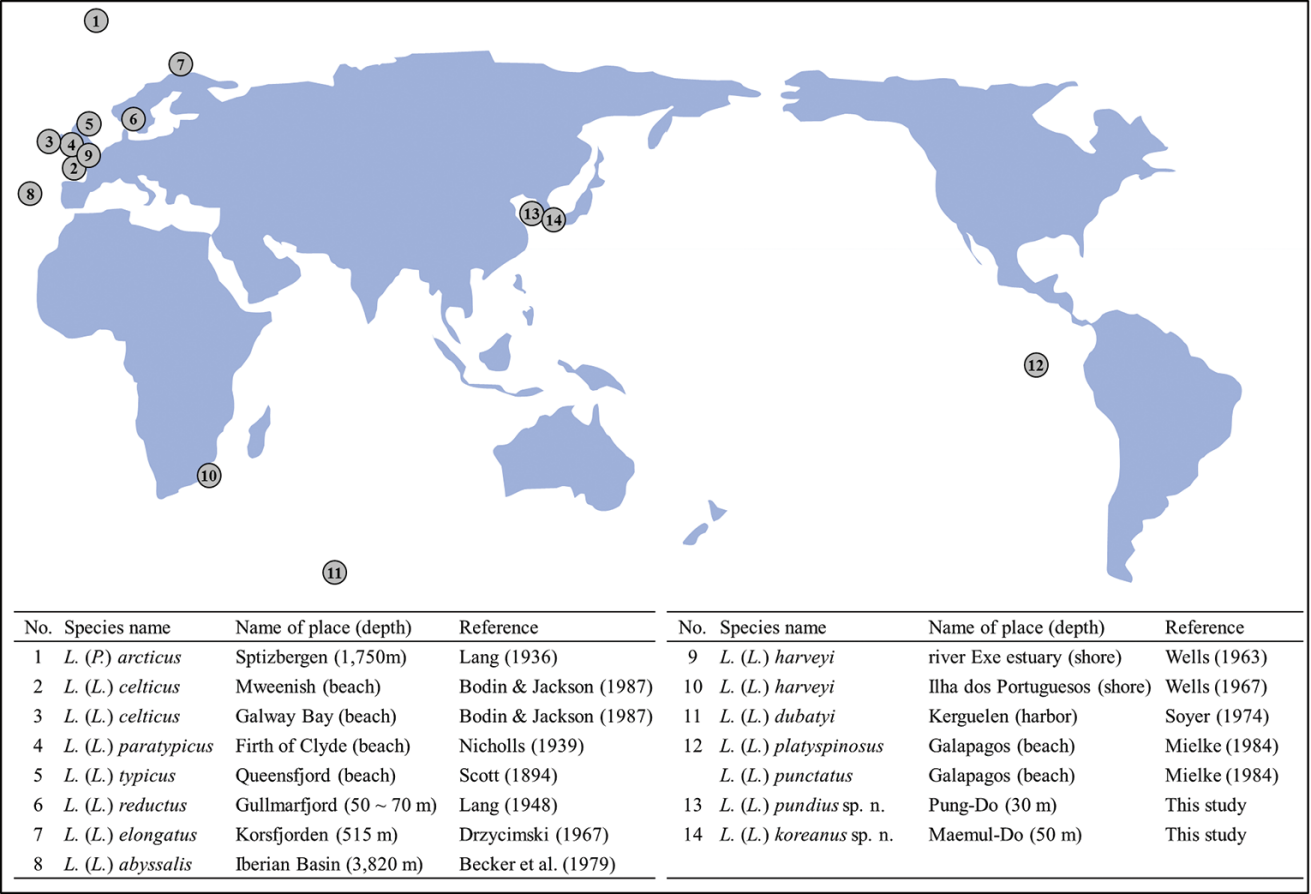
Distribution of genus *Leptopsyllus* and number of specimens based on original papers.

Until now, many species belonging to genus *Leptopsyllus* have been found mainly in Europe in Atlantic Ocean (Fig. 9). Most species are distributed along the coast including islands intertidal zone, but three species, Leptopsyllus (Paraleptopsyllus) arcticus (Lang, 1936), L. (L.) elongates Drzycimski, 1967, and L. (L.) abyssalis Becker, Noodt & Schriever, 1979, were collected from deep sea (deeper than 200 m). Belonging to the family Paramesochridae, species of *Leptopsyllus* are well known for living in sandy bottom, however, some species were found in muddy sediment (Lang, 1948; Drzycimski, 1967; Plum and George, 2009). In this study, L. (L.) pundius sp. n. is found in the sandy sediment, while L. (L.) koreanus was collected from muddy sand sediment. In conclusion, species belonging to the genus *Leptopsyllus* are thought to inhabit a variety of sediments and depths.

### Relationships between two new species and their congeners

The two new species are placed in the genus *Leptopsyllus* T. Scott, 1894 based on the absence of an endopod on P2 and P3, P4 endopod armed with one apical seta, and presence of two setae/spines on the distal segments of the exopods on P2–P4. The two new species are placed in subgenus Leptopsyllus on account of the absence of P3 endopod because the discrepancy in the diagnostic characters between the subgenera *Leptopsyllus* T. Scott, 1894 , and *Paraleptopsyllus* Lang, 1944 is presence or not of P3 endopod.

Leptopsyllus (L.) pundius sp. n. is closely related to L. (L.) punctatus Mielke, 1984 based on combination of three characters: (1) body length, (2) one segment of A2 exopod with 4 setae, (3) P5 exopod separated with the baseoendopod, and 4) the baseoendopod well developed armed with 2 setae (Table 1). However, L. (L.) pundius sp. n. can be differentiated from the congener by (1) the one-segmented mandibular exopod armed with two setae; whereas L. (L.) punctatus has four setae on the exopod of the mandible (Table 1), (2) P6 with one element, and (3) caudal seta III is stout, decorated with spinules. In addition, the structure of the rostrum with a pair of small sensillae of the new species is also unique character in the genus *Leptopsyllus*. Several species, (*Caligopsyllus primus* Kunz, 1975, *Diarthrodella ergeneae* Sonmez, Karaytug & Sak, 2015, *Emertonia clausi* Pointer & Veit-Köhler, 2013, L. (L.) punctatus, L. (L.) pundius sp. n.; L. (L.) koreanus sp. n., and Scottopsyllus (S.) praecipuus Veit-Köhler, 2000) in Paramesochridae have a couple of sensillae on the rostrum. However, rostrum structure in other congeners of the genus *Leptopsyllus* was ignored or is unknown due to the small size of these species.

**Table 1.** Morphological characters of Leptopsyllus (Leptopsyllus). Four groups were distinguished by the feature of the female P5.

Group	Species name	Body Size	A2	Swimming legs								P5	
				P1	P2		P3	P4					
		Female (Male)	No. of exp seg. (total No. of setae)	Enp-2	Exp-1	Exp-2	Exp-2	Exp-2	Exp-3	Enp-1	Enp-2	Exp. separation	Benp. develope (No. of setae)
1	*abyssalis*	730	1(5)	011	0	021	021	021	·	010	·	F	N(0)
	*reductus*	500	?(4)	011	0	0	0	?	?	0	010	F	N(0)
2	*platyspinosus*	400–440 (360)	1(4)	011	0	0	0	0	011	010	·	S	N(0)
3	*celticus*	380–400	1(4)	011	0	1	1	0	021	010	·	F	D(2)
4	*dubatyi*	400–445	2(4)	011	0	0	0	0	011	010	·	S	D(0)
	*harveyi*	420	1(3)	011	0	0	0	0	021	010	·	S	D(0)
	*punctatus*	290–390 (280–380)	1(4)	011	0	0	0	0	011	010	·	S	D(2)
	*typicus*	700	1(4)	011	?	?	0	0	011	0	010	S	D(2)
	*pundius* sp. n.	390	1(4)	011	0	0	0	0	011	010	·	S	D(2)
Unknown	*elongatus*	(900)	1(5)	010	0	0	0	0	011	010	·	?	?
	*paratypicus*	(360)	1(4)	011	1	1	1	0	011	010	·	male only	male only
	*koreanus* sp. n.	(575)	1(4)	010	0	0	0	0	011	010	·	male only	male only

* No.: number, seg.: segment, S: separated, F: fused, D: developed, N: not developed, Unknown : P5 of female were not described in the species of the group

The description of L. (L.) koreanus sp. n. is based on a single male specimen. Although we are not able to compare L. (L.) koreanus sp. n. with its congeners based on female characters, the new species has clear morphological differences from its congeners: (1) single seta on the second segment of the P1 endopod; only one species in the subgenus Leptopsyllus, L. (L.) elongates Drzycimski, 1967, shares this character with L. (L.) koreanus, (2) a single seta on the baseoendopod of P5 in male; this is a unique character in the subgenus Leptopsyllus, and (3) one-segmented endopod of the mandibular palp; L. (L.) koreanus sp. n. shares this character with L. (L.) platypinosus Mielke, 1984. The caudal ramus in the genus *Leptopsyllus* is conical and its distal margin is bluntly pointed; however, caudal seta formula varies among congeners. Leptopsyllus (L.) koreanus sp. n. clearly has caudal seta I, although this seta is obscure in other congeners. Both new species have a tri-articulated seta VII, while L. (L.) punctatus and L. (L.) platyspinosus have a bi-articulated seta VII arising from a chitinous outgrowth on the dorsal surface. However, the caudal rami and setae of other species in *Leptopsyllus* have not been described in detail in other previous studies.

Four distinctive groups within the genus *Leptopsyllus* can be recognized based on the shape of the female P5 (Table 1): (1) the exopod and baseoendopod fused without developed baseoendopodal lobes (L. (L.) abyssalis Becker, Noodt & Schriever, 1979 and L. (L.) reductus Lang, 1948), (2) the exopod and baseoendopod not fused, without developed endopodal lobes (L. (L.) platyspinosus), (3) the exopod and baseoendopod fused, with developed endopodal lobe (L. (L.) celticus Bodin & Jackson, 1987), (4) the exopod and baseoendopod not fused, with developed endopodal lobes (L. (P.) arcticus (Lang, 1936), L. (L.) typicus T. Scott, 1894, L. (L.) dubatyi Soyer, 1974, L. (L.) harveyi Wells, 1963, L. (L.) punctatus, and L. (L.) pundius sp. n.). In the case of the seta formula of P5 exopod, all female species in the genus have three setae. Unfortunately, Leptopsyllus (L.) elongatus, L. (L.) paratypicus Nicholls, 1939, and L. (L.) koreanus sp. n. are cannot be assigned to any of the four groups because they have been described based on the male specimen. More studies will be necessary to prove the relationship among four groups including female and male P5.

### Key to the species of the genus *Leptopsyllus*

The latest key proposed by Bodin & Jackson (1987) includes nine species of *Leptopsyllus*, and does not include the two species L. (P.) arcticus and L. (L.) abyssalis. Because only the males of some species have been described, the extent of sexual dimorphism in mouthparts or P1–P4 was not known. An updated key is developed on the basis of selected characteristics from the original description that identifies species within the genus *Leptopsyllus*.

**Table:** 

1	P3 endopod 1-segmented	**(Subgenus Paraleptopsyllus)**...L. (P.) ***arcticus***
–	P3 endopod absent	**(Subgenus Leptopsyllus)**...**2**
2	P2–P4 exopod 2-segmented	L. (L.) ***abyssalis***
–	P2–P4 exopod 3-segmented	**3**
3	Distal segment of P1 endopod with 1 seta	**4**
–	Distal segment of P1 with 2 elements	**5**
4	A2 exopod with 5 setae; caudal ramus with 5 setae	L. (L.) ***elongatus***
–	A2 exopod with 4 setae; caudal ramus with 7 setae	L. (L.) ***koreanus* sp. n.**
5	Middle segments of P2 and P3 exopod with 1 inner element	**6**
–	Middle segment of P2 and P3 exopod without inner element	**7**
6	Male exopod of P5 with 3 setae	L. (L.) ***paratypicu*** *s*
–	Male exopod of P5 with 4 setae	L. (L.) ***celticus***
7	A2 exopod 2-segmented	L. (L.) ***dubatyi***
–	A2 exopod 1-segmented	**8**
8	A2 exopod with 3 setae	L. (L.) ***harveyi***
–	A2 exopod with 4 setae	**9**
9	P5 endopodal lobe flattened	**10**
–	P5 endopodal lobe well developed	**11**
10	P5 exopod separated, caudal seta III modified	L. (L.) ***platyspinosus***
–	P5 exopod fused with baseoendopod	L. (L.) ***reductus***
11	Each P5 baseoendopodal lobe divided in middle; P1 exp-1 with 2 outer setae	L. (L.) ***typicus***
–	Each P5 baseoendopodal lobe without median incision; P1 exp-1 with 1 outer seta	**12**
12	Caudal seta III stout and decorated with spinules defined at base; each side of P6 with 1 seta	L. (L.) ***pundius* sp. n.**
–	Caudal seta III cylindrical, decorated with long spinules; each side of P6 with 3 setae	L. (L.) ***punctatus***

## Supplementary Material

XML Treatment for Leptopsyllus (Leptopsyllus)

XML Treatment for Leptopsyllus (Leptopsyllus) pundius

XML Treatment for Leptopsyllus (Leptopsyllus) koreanus
